# Stress for invasion success? Temperature stress of preceding generations modifies the response to insecticide stress in an invasive pest insect

**DOI:** 10.1111/eva.12001

**Published:** 2012-09-07

**Authors:** Saija Piiroinen, Anne Lyytinen, Leena Lindström

**Affiliations:** Centre of Excellence in Biological Interactions Research, Department of Biological and Environmental Science, University of JyväskyläJyväskylä, Finland

**Keywords:** adaptive phenotypic plasticity, carry-over, cross-generational effect, invasive species, pyrethroids, species range, stress tolerance, sub-lethal effects

## Abstract

Adaptation to stressful environments is one important factor influencing species invasion success. Tolerance to one stress may be complicated by exposure to other stressors experienced by the preceding generations. We studied whether parental temperature stress affects tolerance to insecticide in the invasive Colorado potato beetle *Leptinotarsa decemlineata*. Field-collected pyrethroid-resistant beetles were reared under either stressful (17°C) or favourable (23°C) insecticide-free environments for three generations. Then, larvae were exposed to pyrethroid insecticides in common garden conditions (23°C). Beetles were in general tolerant to stress. The parental temperature stress alone affected beetles positively (increased adult weight) but it impaired their tolerance to insecticide exposure. In contrast, offspring from the favourable temperature regime showed compensatory weight gain in response to insecticide exposure. Our study emphasizes the potential of cross-generational effects modifying species stress tolerance. When resistant pest populations invade benign environments, a re-application of insecticides may enhance their performance via hormetic effects. In turn, opposite effects may arise if parental generations have been exposed to temperature stress. Thus, the outcome of management practices of invasive pest species is difficult to predict unless we also incorporate knowledge of the evolutionary and recent (preceding generations) stress history of the given populations into pest management.

## Introduction

Invasion of alien species to novel environments poses serious threats to biodiversity, ecosystem function, agriculture and to the health of humans and animals (Mack et al. 2000; Pimentel et al. 2000). Identifying ecological and genetic attributes of invasions will help us to understand the invasion process and contribute to the development of prevention practices (Kolar and Lodge 2001; Lee 2002). As invasive species spread to new environments, they face various natural and anthropogenic stressors such as extreme temperatures or insecticides as well as stress imposed by biotic factors (e.g. competition) that may slow down or limit further spread (e.g. Lee et al. 2003, 2009). Stress tolerance and adaptation to stressful environments are considered to be among the most important factors affecting the invasion success of invasive species (e.g. Sexton et al. 2002; Lee et al. 2003; Lee and Gelembiuk 2008; Weir and Salice 2012). For instance, it is suggested that those populations that originate from variable environments, that is, are tolerant to stressors, are more likely to become invasive than those from more stable conditions (Lee and Gelembiuk 2008). This is because high tolerance may enable organisms to persist under the new environmental conditions and allow enough time for adaptation to occur (Sexton et al. 2002). Ability to physiologically tolerate and adapt to several stressors is therefore important for determining invasive species range limits (Addo-Bediako et al. 2000; Lardies et al. 2004; Gilchrist et al. 2008).

Although stress is considered to play a significant role in species invasions, only few experimental studies have experimentally investigated the short-term responses of organisms to stressors and how these responses can affect their invasion potential (Sexton et al. 2002; Lee et al. 2003; Weir and Salice 2012). The most likely reason for this is because most of the invasion studies are conducted long after the initial invasion has taken place (Puth and Post 2005). Best examples explicitly linking stress effects with invasions mainly come from invasive plants. In plants, plasticity as a means of tolerance to stress is thought to increase invasion success (e.g. Sexton et al. 2002; Richards et al. 2006). The influence of cross-generational stress effects on species invasions has received even less attention (Duckworth 2009; Dyer et al. 2010). It has been documented to have a role in some invasive plant species. For example, exposure of parental generations of *Aegilops triuncialis* annual grass to stressful soils resulted in larger, more fecund progeny, which is a major factor contributing to the spread of the species (Dyer et al. 2010). As for animals, cross-generational (maternal) effects associated with range expansions have been studied in few species (Duckworth 2009 and references there in).

As many invasive insect species are also pests, they are repeatedly exposed to insecticides that incur stress. Adaptation especially to human-altered anthropogenic stress (like insecticides) could be a significant factor promoting pest species invasions (Hufbauer et al. 2012). The question of how invasive species respond and evolve to the management practices is of importance not only in the context of the lethal effects of insecticides and resistance evolution (e.g. McKenzie 2001; ffrench-Constant et al. 2004), but also in the context of the stressful, sub-lethal effects of insecticides (Morse 1998; Cutler et al. 2005). Exposure to insecticide stress (or other kind of stress) may increase phenotypic variation by inducing variable responses in physiological and life-history traits (e.g. Bernard and Lagadic 1993; Hardin et al. 1995; Maltby 1999; Desneux et al. 2007; Talloen et al. 2009). Although most stress responses have negative effects, some may be advantageous, increasing fitness (Magiafoglou and Hoffmann 2003). This latter phenomenon is also known as hormesis, where a mild exposure to a stressor induces beneficial effects but is toxic at higher exposure levels (Morse 1998; Costantini et al. 2010). Mechanisms of hormesis are not very well known, but they can operate through both physiological and epigenetic effects. In the short-term, beneficial plastic responses may enable invasive pest population to persist under unfavourable conditions until genetic adaptation takes place (Sexton et al. 2002). At the same time, if stress responses have a genetic basis, they may enhance rapid adaptation to stressful environments (genetic assimilation) (Parsons 1991; Hoffmann and Hercus 2000; Badyaev 2005).

Another important attribute involved in invasion success of insects, in particular, is temperature. Consequently, insects that spread across environmental gradients can experience strong selection for increased temperature tolerance (Castaneda et al. 2004). Adaptation to certain temperatures, however, may result in reduced fitness in another temperature (Partridge et al. 1995). Moreover, evolutionary responses to one type of stress can result in correlated responses to other stressors (e.g. Hoffmann and Parsons 1989) that can either be negative (trade-offs) or positive (cross-resistance) because many traits are genetically correlated.

Studies on the stress tolerance usually have investigated one stressor at a time. Under natural conditions, insect populations are very likely to experience several stressors either repeatedly or simultaneously, such as predation and insecticides (Campero et al. 2007), and the combined effects of multiple stressors could be more harmful than those attributed to one stressor (Sih et al. 2004; Campero et al. 2007; Coors and De Meester 2008). Furthermore, the response to stress may be influenced by stress/environmental conditions experienced by the previous generations (Mousseau and Fox 1998; Bonduriansky and Day 2009). For example, temperature can have cross-generational effects affecting life-history and physiological traits of the progeny (Crill et al. 1996; Magiafoglou and Hoffmann 2003; Steigenga and Fischer 2007).

Despite the fact that many invasive pest species have expanded their range across changing thermal conditions and are repeatedly controlled by insecticides, combined effect of several stressors or the importance of cross-generational effects of such stressors on life-history and physiological traits has rarely been explored in the context of invasion biology or pest management. Furthermore, global climate change has been predicted to increase temperature variations (e.g. Easterling et al. 2000) suggesting that successive generations of pest populations can experience very different temperature conditions adding importance to the role of thermal stress on pest insect invasions. Therefore, to better understand and predict pest species invasions, it is important to take into account the past and recent history of invasive populations (Roderick and Navajas 2003).

Our study species, the Colorado potato beetle *Leptinotarsa decemlineata* Say, is an invasive pest of potato *Solanum tuberosum* (Casagrande 1987). It is native to Mexico, has spread to the United States and Europe (excluding the United Kingdom and Scandinavia) and is currently spreading to higher latitudes in Europe (Grapputo et al. 2005; EPPO 2006; Boman et al. 2008). The control of this species, as also in many other pest species, relies on the use of insecticides. Consequently, many populations have evolved resistance (Alyokhin et al. 2008). Depending on its origin, the invading population that arrives to a new area can already be resistant to commonly used insecticides. It follows that, for already resistant populations, insecticide treatment is more likely to incur stress instead of lethal effects. Considering the widespread problem of resistance among agricultural pest species (see e.g. Whalon et al. 2008), the consequences of insecticide stress on already resistant populations have not been studied extensively. To investigate separate and combined effects of insecticide stress and thermal stress history on life-history traits, we took advantage of the fact that some beetle populations are already resistant to pyrethroid insecticides. We maintained parental beetle generations for three generations under stressful (low) and favourable (high) temperature regimes, both in the absence of insecticide exposure. We then exposed descendant larvae to the pyrethroid insecticide in a common garden experiment (under 23°C). This is a typical scenario where pest species in newly invaded area are not immediately exposed to insecticides because of a low detection threshold (Lockwood et al. 2007). But when invaded individuals are later detected, they are treated with insecticides to which they can already be resistant. We predicted that past history of thermal stress or current insecticide stress alone would have harmful effects in the form of decreased adult weight, prolonged development time and decreased ability to gain lipid (energy) reserves (Quinlan and Gatehouse 1981; Tan 1981; Bernard and Lagadic 1993; Wang et al. 2009), and that the effect of insecticide stress would be more pronounced in the beetles descendant of parents reared under the stressful temperature environment (Sih et al. 2004). The traits we investigated are all influential for the overwintering survival of these beetles (Hahn and Denlinger 2007; Piiroinen et al. 2011), thus negative effects of stressors may particularly influence the invasion success of the *L. decemlineata*.

## Materials and methods

### Study animals and rearing

Colorado potato beetles used in this study were collected in potato fields in Poland (Bonin, 54°09′N, 16°15′E) (*n* = 200 females) in June 2003. One beetle from each potato plant was collected. Sampled plants were located at least 5–10 m apart. The Polish populations have been heavily controlled by pyrethroids, and the prevalence of pyrethroid resistance in this population is high [99.1% carry a point mutation in the gene *LdVssc1*, which is the major gene involved in pyrethroid resistance (Argentine et al. 1995), unpublished data A. Grapputo, A. Lyytinen and L. Lindström]. Field-collected beetles were mated in the laboratory, and the offspring that hatched were equally subdivided into two fluctuating temperature regimes: stressful, with mean temperature of 17°C (13°C for 4 h, 20°C for 16 h), and more favourable, with mean temperature of 23°C (18°C for 4 h, 25°C for 16 h). The low temperature regime where the temperature decreases to 13°C for 4 h can indeed be considered stressful for the beetles (see also Ferro et al. 1985): Larval-to-adult survival for beetles originating from the same region as in our study was about 50% under 17°C compared to 75% survival under a mean temperature of 23°C (Boman et al. 2008). Moreover, beetle larvae that were kept under average temperature of 13°C suffered over 90% mortality (Lyytinen et al. 2009). The high-temperature regime (mean 23°C) mimics a warm summer (June–August) in Poland (World Weather Information Service). Two replicates for each treatment were maintained in two controlled environmental chambers (Type B1300; Weiss technic, Reinkirchen-Lindenstruth, Germany) for three generations without exposing larvae to insecticides before the start of the experiment. The photoperiod was L:D 16:4 h with crepuscular light 2 h, imitating sunrise and sunset. In each of the three generations, there were a maximum of 50 (a minimum of 17) families per replicate. Because beetles lay fewer eggs under 17°C than under 23°C, all adults during breeding were kept in conditions similar to the experimental individuals to acquire enough offspring for each generation. Each mating pair, that is, family, was reared in a Petri dish lined with a moisturized filter paper and fed with fresh potato leaves (Van Gogh) supplied daily. Eggs of each female were reared to larvae in separate Petri dishes. To allow pupation, last instar larvae were placed in a plastic container filled with peat. After emergence, the adult beetles were reared individually. To allow overwintering (for 8–9 months), an adult beetle was placed in a plastic container filled with peat and fed until it burrowed into the soil. Overwintering conditions were 5°C, dark, and all individuals were kept mixed in the same conditions. The fourth generation beetles used in this study were reared from eggs to 10-day-old adults as described earlier but at a constant temperature of 23°C in a third chamber (Type B1300).

### Insecticide treatment for larvae

Under typical field conditions in Central Europe, larval stages are most likely exposed to an insecticide treatment once in one summer season (Wegorek et al. 2002). To mimic this, larvae were treated with an insecticide once. Three to five 3-day-old larvae of each family (15–17 families in each parental temperature replicate) from the same egg clutch were divided to control and insecticide treatment groups. In total, we evaluated 302 and 315 larvae from the stressful (mean 17°C) and favourable (mean 23°C) parental temperature regimes, respectively. In the insecticide treatment, 1 mL of 3.18 mg/L deltamethrin solution (Trademark Decis; Aventis CropScience, Copenhagen, Denmark) was added to filter paper in a petri dish and let to absorb. This concentration is equivalent to the concentration instructed by the manufacturer and is applied in management practices of the beetle. Larvae were then placed on a potato leaflet in the petri dish. One millilitre of water was used in the control treatment. After 24 h, the survival of the larvae was observed. The surviving larvae were carefully moved to a new petri dish with fresh potato leaflets and reared to adulthood at 23°C. Larvae-to-adult survival and development times were recorded. Newly emerged adults were sexed and weighed (to 0.001 g; Mettler AM100, Columbus, OH, USA).

### Lipid content measurement

To investigate whether insecticide exposure had an effect on energy reserves, the lipid content was measured (see Östman 2005) from 10-day-old adult beetles (total *n* = 264). By this age, the increase in body mass has levelled off and beetles are also already prone to enter diapause (Piiroinen et al. 2010). Energy reserves for overwintering are mainly situated in the fat body of the beetle [over 90% of lipids are storage fat (Lehmann et al. 2012)]. Beetles were first weighed and killed by decapitation. The elytra and legs were carefully removed, and the remainder of the beetle (abdomen and thorax) was weighed before drying at 55°C for 72 h, after which the dry weight was measured. Chloroform–methanol solution (1:1) was used to extract lipids (72 h), and after drying for 72 h, the lipid-free weight of the beetle was measured. The lipid content was calculated by subtracting the lipid-free weight from the dry weight, and the relative lipid content is presented as proportional to dry weight.

### Statistical analyses

Larval survival (dead, alive) and larval-to-adult survival were analysed with a generalized linear mixed effects model (GLMM) with a logit link function in R (R Development Core Team 2011) using the package lme4 (Bates et al. 2011). Models were fitted using Laplace estimation where parental temperature regime and insecticide stress were entered as fixed variables. Replicate (within parental temperature regime) was considered as a random factor. Emergence weight (mg), development time (days) and relative lipid content (proportional to dry weight) were analysed with a nested mixed-effect anova in SPSS (v16; SPSS Inc., Chicago, IL, USA). Sex, insecticide treatment and parental temperature regime were entered as fixed factors, and replicate, considered as a random factor, was nested within parental temperature regime. In the models, we first included all interaction terms between fixed factors and an interaction between the random factor and insecticide treatment. Non-significant interactions (*P* > 0.05) were omitted from the final models (Sokal and Rohlf 2003). As we were interested in whether response to larval insecticide treatment differs between parental temperature regimes, the interaction between these two variables was retained in the final models. Emergence weight was log transformed, and the development time was transformed to ranks (mean was used to ties) to meet the assumptions of parametric statistics. Relative lipid content was transformed by taking the arcsine and square root of the observed proportions.

## Results

### Survival after insecticide treatment

**Figure 1:**
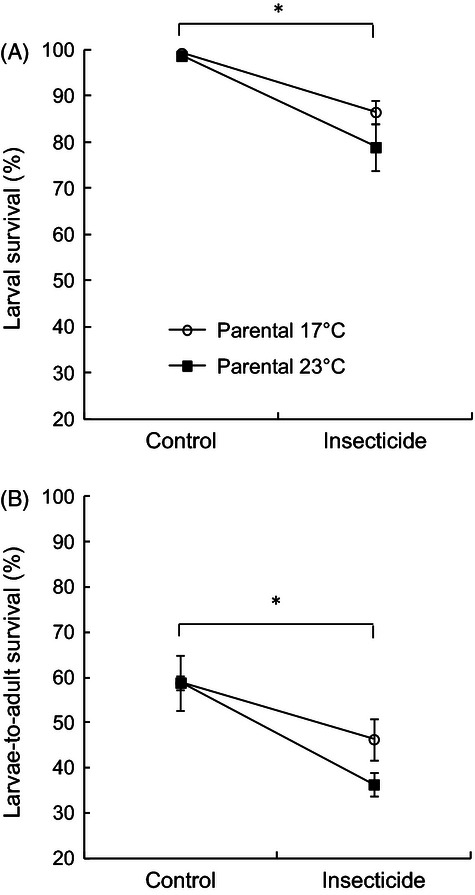
Immediate larval survival (% ± SE) (A) and larval-to-adult survival (B) for control and insecticide-stressed Colorado potato beetles from stressful (17°C) and favourable (23°C) parental temperature regimes. Replicates are pooled in the figure. Insecticide treatment reduced significantly both immediate and larval-to-adult survival as indicated by an asterisk.

Insecticide treatment reduced larval survival on average 17.3% (±2.2 SE) (GLMM, *z* = −3.06, *P* = 0.002, Fig. 1A). The high larval survival (82.7%) after exposure to a pyrethroid insecticide concentration, which is used in management procedures and is specifically efficient against small larvae, confirms that the beetle population used in this experiment was resistant to pyrethroid insecticides. For comparison, a milder concentration (0.8 mg/L) resulted in only 20% survival in a susceptible Russian population (Piiroinen 2010). Larval survival after 24 h of exposure to insecticide was not affected by the parental temperature regime (17°C vs 23°C, *z* = −0.54, *P* = 0.593). The effect of insecticide treatment on larval survival was not dependent on the temperature at which the parental generations were reared (insecticide stress × parental temperature regime: *z* = 0.10, *P* = 0.921).

### Larval-to-adult survival

Larvae-to-adult survival was on average 41.2% (±2.8 SE) in the insecticide treatment and 59.2% (±2.8 SE) in the control (*z* = −2.17, *P* = 0.029, Fig. 1B). The respective values excluding larvae that died within 24 h of exposure were 49.8% (±3.1 SE) and 59.7% (±2.8 SE). Larvae-to-adult survival was not affected by parental temperature regime (*z* = 0.01, *P* = 0.995). The effect of insecticide treatment on larvae-to-adult survival was not dependent on the temperature at which the parental generations were reared (insecticide stress × parental temperature regime: *z* = −1.27, *P* = 0.205). The difference in larvae-to-adult survival (9.6%) (excluding larvae that died within 24 h) between control and insecticide-treated beetles was smaller in magnitude than in the larval survival after 24 h of exposure (17.3%).

### Life-history and physiological traits

**Figure 2:**
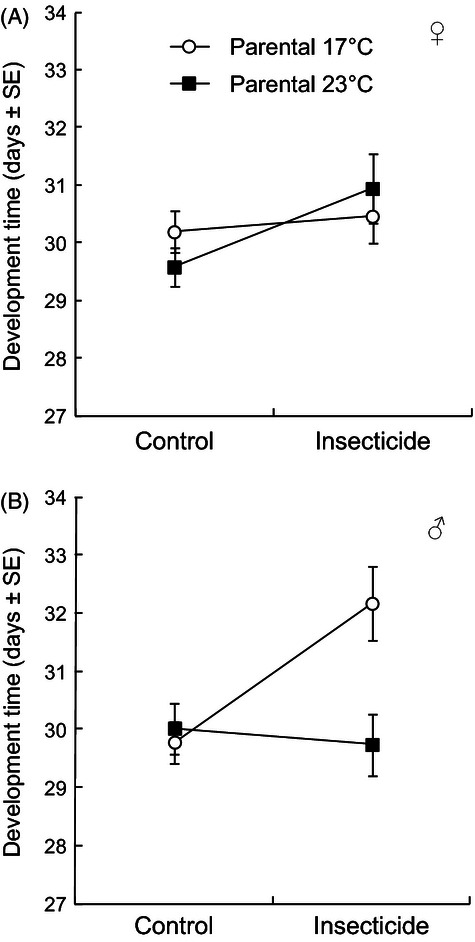
Development time (days ± SE) for control and insecticide-stressed (A) female and (B) male Colorado potato beetles from stressful (17°C) and favourable (23°C) parental temperature regimes. Replicates are pooled in the figure. A significant interaction between insecticide treatment and parental temperature regime was observed for males (B) (Table 1). Insecticide treatment delayed male development time when the parental generations had been reared under the stressful temperature regime but not when males were descendants of parents reared under the favourable temperature regime.

Egg-to-adult development time was negatively correlated with adult emergence weight (Spearman's rho, Females: *r* = −0.28, *P* < 0.001, *n* = 160; Males: *r* = −0.31, *P* < 0.001, *n* = 146). In other words, beetles whose development time was longest were also smallest. The effect of insecticide treatment was dependent on both sex and parental temperature regime (nested mixed effect anova, 3-way interaction: *F*_1,296_ = 5.62, *P* = 0.018). Thus, sexes were analysed separately. In females, development time was not affected by insecticide exposure, parental temperature regime or replicate (nested within parental temperature regime) (Table 1, Fig. 2A). The effect of insecticide treatment on development time was also not dependent on the temperature regime at which the parental generations were reared (Table 1, Fig. 2A). In contrast, in males, there was a significant interaction between insecticide exposure and parental temperature regime (Table 1), indicating that the effect of insecticide treatment on development time differed between parental temperature regimes. Insecticide treatment delayed male development time when the parental generations had been reared under the stressful (mean 17°C) temperature regime (mean_Insecticide_ ± SE: 32.17 ± 0.63 vs mean_Control_ ± SE: 29.78 ± 0.36 days) but not when males were descendants of parents reared under the favourable (mean 23°C) temperature regime (mean_Insecticide_ 30.02 ± 0.44 vs mean_Control_ 29.74 ± 0.53 days, Fig. 2B). Non-insecticide-exposed males from the stressful and favourable parental temperature regimes had similar development times (Fig. 2B). There were no differences between replicates (within parental temperature regime) in male development time (Table 1).

**Table 1:** Results of a nested mixed-effect anova investigating the effect of insecticide stress and parental rearing temperature on development time. Males and females were analysed in separate models. Replicate was treated as a random factor

Sex	Effect	df	MS	*F*	*P*
Female	Insecticide treatment	1	2466.84	1.18	0.279
	Parental temperature regime	1	138.76	0.05	0.842
	Replicate (within parental temperature regime)	2	2735.91	1.31	0.273
	Insecticide treatment × Parental temperature regime	1	2032.95	0.97	0.326
Male	Insecticide treatment	1	8212.23	4.96	0.027
	Parental temperature regime	1	6065.24	7.84	0.068
	Replicate (within parental temperature regime)	2	691.64	0.42	0.659
	Insecticide treatment × Parental temperature regime	1	12783.22	7.73	0.006

**Figure 3:**
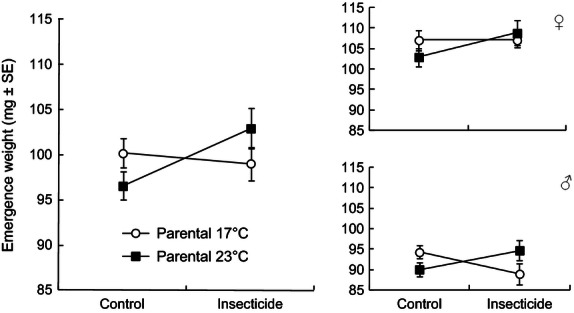
Emergence weight (mg ± SE) for control and insecticide-stressed Colorado potato beetles from stressful (17°C) and favourable (23°C) parental temperature regimes. Replicates are pooled in the figure. There was a significant interaction between insecticide treatment and parental temperature regime (Table 2). Insecticide exposure did not affect emergence weight when the parents had been reared under the stressful temperature regime, but it increased emergence weight when beetles were descendants of parents that had experienced the favourable temperature regime. Non-insecticide-exposed beetles from the stressful parental temperature regime had higher emergence weight than those descending from parents that had experienced the favourable temperature regime.

The effect of insecticide exposure on emergence weight differed between parental temperature regimes shown by a significant two-way interaction (Table 2a). Insecticide exposure did not affect emergence weight when the parental generations had been reared under the stressful temperature regime (mean_Insecticide_ ± SE: 99.05 ± 1.84 vs mean_Control_ ± SE: 100.18 ± 1.61 mg), but it increased emergence weight when beetles were descendants of parents that had experienced the favourable temperature regime (mean_insecticide_ 102.93 ± 2.21 vs mean_Control_ 96.49 ± 1.58 mg, Fig. 3). Non-insecticide-exposed beetles whose parents had been reared under the stressful temperature regime had higher emergence weight than those descending from parents that had experienced the favourable temperature regime (100.18 vs 96.49 mg). Females were heavier than males (Table 2a). There were no differences between replicates (within parental temperature regime) in emergence weight (Table 2a).

**Table 2:** Results of a nested mixed-effect anova investigating the effect of insecticide stress and parental rearing temperature on (a) emergence weight and (b) relative lipid content (proportional to dry weight). Replicate was treated as a random factor

Effect	df	MS	*F*	*P*
(a) Emergence weight				
Insecticide treatment	1	0.002	0.57	0.450
Parental temperature regime	1	<0.001	0.07	0.809
Sex	1	0.283	80.60	<0.001
Replicate (within parental temperature regime)	2	0.004	1.20	0.303
Insecticide treatment × Parental temperature regime	1	0.019	5.48	0.020
(b) Relative lipid content				
Insecticide treatment	1	0.01	0.81	0.369
Parental temperature regime	1	0.07	13.78	0.049
Sex	1	0.01	0.93	0.337
Replicate (within parental temperature regime)	2	0.01	0.50	0.609
Insecticide treatment × Parental temperature regime	1	0.03	3.36	0.068

**Figure 4:**
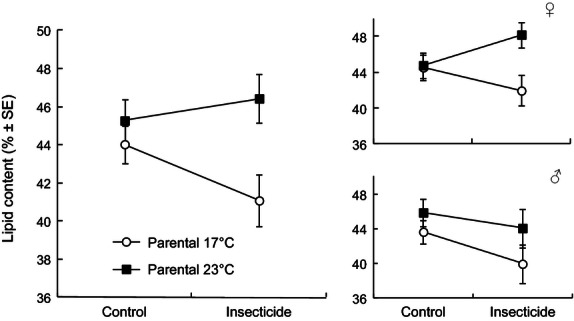
Relative lipid content (% dry weight ± SE) for control and insecticide-stressed Colorado potato beetles from stressful (17°C) and favourable (23°C) parental temperature regimes. Replicates are pooled in the figure. There was a marginally significant interaction between insecticide treatment and parental temperature regime (Table 2b). Insecticide treatment tended to decrease relative lipid content when the parental generations had been reared under the stressful temperature regime but not when reared under the favourable temperature regime.

There was a marginally significant interaction between insecticide exposure and parental temperature regime (Table 2b) indicating that the effect of insecticide exposure on relative lipid content may differ between parental temperature regimes. Insecticide treatment tended to decrease relative lipid content when the parental generations had been reared under the stressful temperature regime (mean_Insecticide_ 41.08 ± 1.35 vs mean_Control_ 44.03 ± 0.98%) but not when reared under the favourable temperature regime (mean_Insecticide_ 46.71 ± 1.28 vs mean_Control_ 45.08 ± 1.03%, Fig. 4). For the beetles from the stressful parental temperature regime, the difference in lipid content stemmed from the trend for individuals exposed to insecticide to be lighter (in dry weight) than those that were not exposed (Three-way mixed-effect anova: *F*_1,133_ = 3.56, *P* = 0.061). As relative lipid content increases with dry weight (Spearman's rho = 0.70, *P* < 0.001, *n* = 263) (i.e. smaller individuals have lower relative lipid content than larger ones), this slight difference in dry weights translated into a larger difference in relative lipid content.

## Discussion

The impetus for our experiment comes from observations that tolerance to various stressors can influence a species' geographical range (see e.g. Lee and Gelembiuk 2008; Kellermann et al. 2009) as well as from recent literature documenting the potential for cross-generational effects to modify on invasion success (Duckworth 2009; Dyer et al. 2010). Here, we conducted an experiment to examine the combined effect of two common stressors on fitness correlates associated with the overwintering survival (Hahn and Denlinger 2007; Piiroinen et al. 2011), of the Colorado potato beetle, an invasive species of worldwide economic importance.

The beetles studied were in general very tolerant to insecticide exposure but the temperature at which insecticide-resistant parents were reared mediated the response of offspring to insecticide exposure. The parental temperature stress alone affected beetles positively (increased adult weight, Fig. 3), but it impaired their tolerance to insecticide exposure (delayed development time in males and decreased lipid content, Figs 2 and 4). In contrast, offspring from the favourable temperature regime reached heavier adult weight in response to insecticide exposure (Fig. 3). In other words, the negative effects manifested themselves only when both the parental temperature stress and insecticide exposure were present. This supports the view that the organism's ability to endure additional stress is decreased under the circumstance of already having experienced other stressors (Sih et al. 2004), which, in our case, would extend to include the stressful temperature regime experienced by the parents. All together, these results indicate that to develop sustainable pest management strategies as well as to understand species invasion success, we cannot ignore the recent (parental temperature stress) history or the evolutionary history (resistance status) of invading pest populations.

There are several potential explanations for the observed differences in stress tolerance between the parental thermal regimes. Firstly, there might have been selection during the experiment (Partridge et al. 1995), which resulted in correlated responses also in tolerance to insecticide stress possibly via genetic correlation. Alternative explanation for the differences could stem from random effects due to the sample size. In this experiment, population sizes were a maximum of 100 reproducing individuals in each replicate and therefore they might have been vulnerable to random changes (Willi et al. 2006). However, most traits (except for emergence weight) did not differ between the replicates of each treatment suggesting that, if present, such random effects were minor. A third and more likely scenario is that the lower parental temperature regime was stressful for the parent beetles (Ferro et al. 1985; Boman et al. 2008; Lyytinen et al. 2009). This then implies negative cross-generational effects on the next generation (Crill et al. 1996; Steigenga and Fischer 2007) decreasing beetles' tolerance to insecticide stress. In general, the parental (mainly maternal) experience of the environment can be transmitted to offspring via cytoplastic egg factors (Mousseau and Fox 1998) but also via epigenetic mechanisms (Angers et al. 2010). An increasing number of studies have indicated that the epigenetic effects can be important in influencing the phenotypes and can also be passed on to the next generation (Bossdorf et al. 2008; Bonduriansky and Day 2009; Angers et al. 2010). It is, however, unclear how parental temperature would affect, in particular, tolerance to insecticide stress.

Whereas most stress responses have negative effects (e.g. Quinlan and Gatehouse 1981; Wang et al. 2009), our results show that the presence of one stress factor, either parental temperature stress (see also Crill et al. 1996; Steigenga and Fischer 2007) or insecticide exposure (Cutler et al. 2005; Cohen 2006), resulted in a beneficial response as shown by higher adult weight in both cases (see Fig. 3). It could be that the parent females lay bigger eggs in response to low temperature (Crill et al. 1996) they had experienced, and this then resulted in heavier adults. The increased adult weight of insecticide-treated beetles from the favourable temperature regime (Fig. 3) could instead suggest that they were able to compensate for the harmful effects of insecticide stress by increasing feeding or assimilation of food (Campero et al. 2007). The compensatory effects probably took place after the exposure to insecticide when treated larvae were fed with insecticide-free leaves as observed in Egyptian cotton leafworm larvae *Spodoptera littoralis* (Bernard and Lagadic 1993). This type of positive cross-generational and within-generational stress effects on the phenotype can play a significant role in species invasions. In plants, for instance, positive cross-generational stress effects have been observed in an annual grass, *Aegilops triuncialis*, where exposure of parental generations to stressful soils resulted in larger, more fecund progeny facilitating invasion to novel environments (Dyer et al. 2010). Similarly, increased root investment in response to cold temperatures has facilitated invasion of a saltcedar *Tamarix ramosissima* (Sexton et al. 2002). Beneficial, hormetic effects in response to insecticides can confer serious problems for pest management practices because they may, for example, give rise to pest outbreaks (Cohen 2006). Pest outbreaks, in turn, can lead to increased use of additional insecticides, worsening the resistance problem. Also, hormetic effects may contribute to the spread of invasive pest species to novel environments by, for example, inducing dispersal activity (Cohen 2006), although direct links between hormesis and pest invasions have not been established.

Our results also demonstrated that male and female Colorado potato beetles responded to stressors differently with males being more sensitive to stress (Fig. 2). Our study is in line with numerous other studies that have also shown sex-specific effects of stress in insects [e.g. mosquitos (Tseng 2004), butterflies (Fischer and Fiedler 2000; Karl et al. 2010)]. Sex differences in tolerance to stressors may be related to sexual dimorphism, for instance, in size, or to the fact that pyrethroid resistance in this species is linked to sex chromosome (Argentine et al. 1995). In general, males have faster development time than females because of protandry selection (maximization of mating opportunities) (Fagerström and Wiklund 1982), and females are heavier as a result of fecundity selection (Roff 2002). Thus, as a result of differential selection pressures between sexes, they may differ in their response to stress owing to differential canalization of traits (Fischer and Fiedler 2000). For management purposes, the fact that males may be more sensitive to stress might not be essential because the population growth rate in polygamous species is generally more dependent on the number of females.

Considering our results in a wider perspective, it should be noted that where beneficial effects of stress are observed in some traits (see also Magiafoglou and Hoffmann 2003; Steigenga and Fischer 2007), there might be costs related to stress that are seen in other traits such as in immune function (Stoks et al. 2006; Costantini et al. 2010). These and our results reflect the fact that many external (e.g. temperature) and intrinsic (e.g. sex) factors influence the response to stress and that these factors can interact in complicated ways. This emphasizes the importance of considering the overall fitness consequences when assessing the influence of stressors on invasion success and pest management outcomes. For example, repeated insecticide stress can increase population growth rate of insect pest populations (Yin et al. 2009). This increase in population size can contribute to higher propagule pressure, which is positively related to invasion success (Memmott et al. 2005). Unfortunately, we were not able to examine whether fitness components measured in the present study would translate into net negative or positive fitness effects because the measurement of lipid content is lethal for the beetles. However, individual-based studies on the effects of stress can help in understanding the population level effects (Maltby 1999). As for the Colorado potato beetle, adult weight is positively associated with overwintering survival (Piiroinen et al. 2011), and this, via changes in population size, can be a crucial factor connected to the successful invasion of new environments.

Despite the fact that numerous pest species populations have evolved resistance to various insecticides creating a serious threat worldwide (see e.g. Whalon et al. 2008), the faith of those individuals that survive the (sub-lethal) insecticide treatment has received surprisingly little attention, most of the studies focusing on mortality factors. Recently, it has been suggested that adaptation especially to anthropogenic stress and human-altered habitats could be a significant factor promoting pest species invasions to other human-altered regions (Hufbauer et al. 2012). From a pest management perspective, the fact that cross-generational stress can modify the response of the beetles in two directions with respect to fitness correlates means that the same management practice may have opposite outcomes depending on the previous stress experienced in preceding generations, that is, recent stress history of the population. These results suggest that a re-application of insecticides on already resistant individuals may result in hormetic effects potentially leading to pest outbreaks and boost invasion potential. As for the Colorado potato beetle, a continued use of pyrethroid insecticides may select for those individuals that will have better chances in overwintering (larger size, see also Piiroinen et al. 2011), potentially increasing the pest problem. Therefore, when beetles invade from areas where they have been repeatedly controlled by pyrethroid insecticides, treating the first invaders with the same insecticide in the newly invaded area is not likely to be the most suitable strategy. On the other hand, our results indicate that if invading populations are already stressed (parental generations), this can impair the pest insects' tolerance to insecticide. Decreased ability of the Colorado potato beetles to gain lipid reserves (Fig. 4), which are the main source of energy during overwintering, may result in higher probability to die during winter (Hahn and Denlinger 2007).

All together, we can conclude that to predict and prevent pest invasions, we need to know not only the resistance status of the invading populations to commonly used insecticides and the most likely invasion routes, but also the recent stress history of the populations. To accomplish this, information exchange of the actual pest control practices (used chemicals etc.), and of the other potential stress factors influencing pest populations, among pest management authorities, farmers and scientific researchers should be augmented. Lastly, our study emphasizes the importance of incorporating evolution as well as plastic responses in pest management of invasive species (Thrall et al. 2011; Hufbauer et al. 2012). Oversimplification of potentially complex responses of organisms to environmental variation and stressors in pest management could lead to management failure of invasive pests, or in the worst case, result in undesired consequences.

## Data Archiving Statement

Data for this study deposited at Dryad- doi:10.5061/dryad.qm009.
